# Rationale for the Use of 8‐Aminoguanine for the Management of Cystitis

**DOI:** 10.1111/iju.70418

**Published:** 2026-06-16

**Authors:** Lori A. Birder, Joel N. H. Stern, Robert Moldwin, Edwin K. Jackson

**Affiliations:** ^1^ Renal‐Electrolyte Division, Department of Medicine University of Pittsburgh School of Medicine Pittsburgh Pennsylvania USA; ^2^ Department of Pharmacology and Chemical Biology University of Pittsburgh School of Medicine Pittsburgh Pennsylvania USA; ^3^ Arthur Smith Institute for Urology, Northwell Health Zucker School of Medicine at Hofstra/Northwell Lake Success New York USA

**Keywords:** 8‐aminoguanine, bladder, cystitis, oxidative stress, purines

## Abstract

Bladder pain and associated lower urinary tract symptoms (LUTSs) are hallmark features of interstitial cystitis/bladder pain syndrome (IC/BPS) and hemorrhagic cystitis (HC). IC/BPS is characterized by bladder‐based pain and irritative bladder symptoms, with or without gross inflammatory changes (Hunner lesions) in the bladder wall, and the absence of well‐described causes such as infection or neoplastic disease. The clinical features of HC are similar to IC/BPS, but in this instance, they are always associated with visible inflammatory disease with known etiologies such as infection or a complication of radiation and/or chemotherapy. While progress has been made in defining the spinal circuits that gate pain signals emanating from the pelvic viscera, lack of a clear understanding of the mechanisms leading to cystitis‐induced visceral pain and lack of validated therapeutic targets for cystitis are a source of frustration for clinicians treating conditions such as IC/BPS and HC and their patients. This review will focus on an emerging therapy for cystitis, namely 8‐aminoguanine (8‐AG), which inhibits the enzyme purine nucleoside phosphorylase (PNPase). PNPase is important for metabolism of “tissue‐protective” purine metabolites into “tissue‐damaging” purines that generate free radicals. IC/BPS patients without and with Hunner lesions exhibit a dysregulation of purine metabolism, with elevated levels of urotoxic purine metabolites compared to healthy controls. Rodents treated with cyclophosphamide are a standard animal model for cystitis and exhibit multiple abnormalities, including increased urinary frequency, neural mechanosensitivity, suppression of pain‐reducing purines, and increased urothelial inflammation/damage. Orally administered 8‐AG prevents all observed histological, structural, biochemical, and physiological abnormalities in cyclophosphamide‐induced cystitis. These findings demonstrate that 8‐AG holds promise for reducing pain and preventing bladder damage for patients with cystitis‐associated pain and LUTS.

## 1 Introduction

Cystitis indicates bladder inflammation which can have different causations though with many shared symptoms. This review will highlight two different forms of abacterial cystitis: interstitial cystitis/bladder pain syndrome (IC/BPS) and hemorrhagic cystitis (HC), the latter often caused by chemotherapy or irradiation. While both may exhibit inflammation, vascular abnormalities, and the loss of permeability of the bladder mucosa, there are differences in terms of underlying etiologies.

A hallmark of multifactorial functional pain syndromes, such as IC/BPS, is pain in the bladder and surrounding pelvic region with an absence of demonstrable pathology of the viscera and associated nerves. IC/BPS predominantly affects women, remains of uncertain etiology, poses diagnostic challenges, and currently lacks a well‐defined therapeutic target. Moreover, existing treatment options offer limited efficacy [1, 2]. IC/BPS is characterized by pelvic pain attributed to the bladder, accompanied by irritative voiding symptoms. Emerging evidence indicates that IC/BPS encompasses a spectrum of etiologies, ranging from localized bladder‐specific (“bladder‐centric”) abnormalities to systemic neurologic or immunologic dysfunctions beyond the bladder. While cystoscopic findings are often unremarkable, approximately 15% of patients exhibit discrete areas of pronounced inflammation known as Hunner lesions (HL) [3, 4]. Regardless of the presence or absence of Hunner lesions, end organ–derived pain remains a prevalent feature of IC/BPS. A urothelial source of nociceptive signaling has been well‐documented, underscoring the bladder epithelium's potential role in symptom generation [5, 6]. While progress has been made to delineate the spinal circuits that gate pain signals emanating from the viscera, lack of a clear understanding of pain mechanisms, associated comorbidities, and a therapeutic target are a source of frustration for the clinician as well as an enormous financial burden for the patient.

HC is a severe inflammatory condition of the lower urinary tract (LUT) that leads to hematuria, blood clots in urine, dysuria, fever, inability to urinate, and loss of bladder control [7, 8]. HC can be caused by toxins, pathogens, and certain medical conditions and is a complication of radiation therapy and chemotherapy, such as with cyclophosphamide (CYP) [9]. For example, in the biotransformation of CYP, nitrogen mustard and acrolein are formed and are highly toxic to the epithelial cells lining the bladder lumen (*urothelium*), which typically exhibit a slow rate of turnover [10, 11]. CYP is commonly used to induce preclinical models of HC, which exhibit multiple abnormalities including increased urinary frequency, neural mechanosensitivity, and increased urothelial inflammation/damage. HC is most common in patients treated with high‐dose CYP in the setting of bone and soft tissue sarcoma and hematopoietic cell transplantation, or as a late toxicity of pelvic radiation therapy when the bladder is within the radiation treatment field [12, 13]. HC occurs in roughly 10% of patients exposed to high doses of CYP chemotherapy or in patients who undergo pelvic irradiation to treat malignancy. HC affects quality of life and poses a substantial burden to the health care system.

## 2 Current Therapeutic Approaches

Current management strategies and therapies for HC include supportive measures (hyperhydration, forced diuresis, and bladder irrigation), intravesical astringents (e.g., the tissue fixative formalin), or the drug sodium 2‐mercaptoethane sulfonate (mesna) [14, 15]. Mesna is typically administered intravenously in three doses: The first dose is given 15 min prior to the chemotherapeutic drug, followed by two doses approximately 3 and 8 h after the initial administration. However, mesna is ineffective as a treatment once HC has developed and is associated with a number of adverse effects. IC/BPS current treatments range from supportive therapies (including physical therapy, dietary changes) and may also include tricyclic antidepressants, antihistamines, pentosan polysulfate, bladder instillations (such as dimethyl sulfoxide, urinary anesthetics, or glycosaminoglycans), endoscopic fulguration and/or steroid injection (Hunner lesions), or neuromodulation [16]. As many promising pharmacologic treatments have not shown effectiveness compared to placebo control, there remains a critical unmet need for safe and effective therapeutics for the treatment of bladder cystitis.

## 3 Purine Metabolism: Protective Roles of Inosine and Damaging Roles of Hypoxanthine

Oxidative stress, characterized by dysregulated elevations in reactive oxygen species (ROS), is well‐known to be a key contributor to the pathogenesis of various inflammatory and tissue‐injurious conditions [17, 18, 19]. Enhanced ROS generation is a common feature of numerous chronic pain states, where it plays a pivotal role in amplifying nociceptive signaling and inflammatory responses. Consequently, targeting ROS and the broader oxidative stress pathways represents a promising therapeutic strategy. Recent findings suggest that dysregulation of purine nucleoside phosphorylase (PNPase) activity contributes to oxidative stress and subsequent cellular injury [20]. PNPase, a member of the glycosyltransferase family, is expressed in both prokaryotic and mammalian systems and plays a pivotal role in the purine salvage pathway [21]. PNPase is important for the metabolism of “tissue‐protective” purine metabolites (inosine and guanosine) into “tissue‐damaging” purines (hypoxanthine [HX] and xanthine) that generate free radicals (e.g., ROS) [22, 23].

Inosine has attracted increasing interest due to its demonstrated anti‐inflammatory and cytoprotective effects across multiple target organ systems. The mechanism of action may involve stimulation of adenosine receptors and prevention of oxidative damage via scavenging of free radicals and peroxynitrite [24, 25]. In addition, inosine has been shown to reduce nociception in chronic neuropathic and inflammatory pain models [25, 26]. In contrast to inosine and guanosine, elevated levels of inosine's downstream metabolite HX over time may exhibit harmful effects due to production of free radicals (superoxide and peroxynitrite) when metabolized by xanthine oxidase (XO) to xanthine and hence to uric acid. It is well‐known that the metabolism of HX to xanthine and xanthine to uric acid by XO produces tissue‐damaging ROS_(specifically, superoxide anion [O^−^_2_] and hydrogen peroxide [H_2_O_2_]) [27]. Increased metabolism of purines has been linked to inflammatory and other disorders including vascular disease and ischemia [28]. For example, it has been established that acrolein, a metabolite of CYP, promotes oxidative stress and cellular damage. Accumulation of oxidative damage over time negatively affects all components of the LUT system (e.g., smooth muscle, nerves, vasculature, and epithelium). Not surprisingly, treatments that inhibit oxidation of HX to xanthine suppress inflammatory cytokines and oxidative stress in many disorders. In this regard, XO inhibitors (which suppress metabolism of HX to xanthine and xanthine to uric acid) are the standard treatment for gout, a disease characterized by inflammation and joint pain [29].

## 4 Abnormal Purine Metabolism as a Significant Contributory Factor in Bladder Cystitis

One of our innovations is the novel concept that bladder cystitis is associated with altered purine metabolism, which involves increased oxidative stress/free radical cellular damage ultimately leading to altered sensation and impaired cellular/neural function and that modulation of PNPase will prevent these changes by protecting mitochondria and/or inhibiting oxidative stress. Our advantage is that the PNPase inhibitor, 8‐aminoguanine (8‐AG), which can be administered orally, treats the underlying cause of these disorders at the cellular level without adverse side effects. 8‐AG acts upon the underlying pathology to prevent cellular function. Preliminary preclinical studies have demonstrated an acceptable safety and toxicity profile, with no detectable adverse effects on critical organ systems.

In a CYP‐induced model of HC, rats exhibited significant pathological changes compared to untreated controls, including: (1) impaired bladder filling, storage, and voiding function; (2) heightened tactile sensitivity to mechanical stimuli; (3) overt inflammatory changes, such as urothelial petechial hemorrhages; (4) elevated expression of biomarkers associated with nociception and oxidative stress; and (5) increased activation of spinal microglia, a key contributor to chronic pain states [20]. Notably, oral administration of the PNPase inhibitor 8‐AG fully reversed these CYP‐induced abnormalities, restoring bladder function and sensory thresholds to levels observed in healthy controls [20]. Furthermore, delayed initiation of 8‐AG treatment—after the onset of CYP‐induced bladder inflammation—significantly attenuated bladder hyperactivity and mechanical allodynia. These findings are particularly compelling, as they suggest that 8‐AG may offer therapeutic benefit even after cystitis has been established. Our findings demonstrate that 8‐AG reverses CYP‐induced increases in urinary frequency, single afferent unit hypersensitivity, and pain‐related behaviors through a pleiotropic mechanism of action. Because PNPase inhibition prevents the metabolism of inosine to HX and guanosine to guanine, the uroprotective effects of PNPase inhibitors—particularly 8‐AG—are likely mediated, at least in part, by increased bladder levels of inosine and guanosine (uroprotective purines) and decreased levels of HX (a urotoxic purine and ROS generator). However, the efficacy of 8‐AG on bladder form and function may extend to pleiotropic effects associated with blocking PNPase including indirectly activating adenosine receptors, increasing tissue/cellular protective purines, and reducing damaging purines thereby reducing sources of ROS and pathways that impact immune function and inflammation.

## 5 Clinical Perspectives: Rationale for Use of 8‐Aminopurines

The current line of preclinical investigation strongly suggests that inhibition of PNPase could reverse or restore cellular functions of inflamed and painful bladder conditions to a healthier state. PNPase inhibitors could be a potential “first‐line” therapy and may also be of benefit for patients with extravesical pain disorders that may also involve oxidative stress and inflammation‐related ROS production (e.g., myofascial pelvic pain, and fibromyalgia). Thus, changes in urotoxic purine products could even represent a contribution due to systemic/central pathologies.

The relationship between chronic pain syndromes, such as IC/BPS, and oxidative stress has been widely described in patients and animal disease models with suggestions that complications may be caused and worsened by ROS. For example, increased expression of inducible nitric oxide synthase (iNOS) is well known to enhance ROS production and appears to be a biomarker for IC/BPS patients with Hunner lesions [19]. Importantly, our published findings suggest that 8‐AG would be a strong candidate for clinical testing. These findings indicate that the urinary purine metabolome is altered in patients with IC/BPS (both with and without Hunner lesions), implicating excessive PNPase activity in the pathophysiology of IC/BPS [30]. As anticipated, we also found significant increases in urinary isoprostanes [30]. These prostaglandin‐like compounds are indices of endogenous oxidative stress [31] and generation of ROS in both IC/BPS patients with and without Hunner lesions. These results are particularly noteworthy because all patients were evaluated during routine clinical care and were receiving a variety of therapies that could potentially have influenced the outcomes. Despite differences in gross cystoscopic findings between the groups, our data suggest the presence of shared underlying pathophysiological mechanisms linking the patient populations included in this study. Our data further suggest that treating such patients with an inhibitor of PNPase could be beneficial by reducing free radical formation and oxidative stress, thereby minimizing tissue damage/inflammation and painful bladder symptoms.

## Author Contributions

All authors contributed to the writing and proofing of the review.

## Conflicts of Interest

L.A.B., A.W.J., and E.K.J. are listed as inventors on PCT application no. 62/877/220. The remaining authors declare no conflicts of interest.
